# Wild‐type transthyretin cardiac amyloidosis and aortic stenosis: Can carpal tunnel syndrome help distinguish the chicken from the egg?

**DOI:** 10.1111/joim.20042

**Published:** 2024-11-28

**Authors:** Marc‐Antoine Delbarre, Gagan Deep Chadha, Mohamed‐Salah Annabi, Refaat Nouri, Amira Zaroui, Paul Blanc‐Durand, Diana Rasolonirina, Mounira Kharoubi, Ancuta Bejan, Arnaut Galat, Silvia Oghina, Philippe Pibarot, Christophe Tribouilloy, Thibaud Damy

**Affiliations:** ^1^ Department of Internal Medicine CHU Amiens Amiens France; ^2^ UR 7517, MP3CV Jules Verne University of Picardie Amiens France; ^3^ Referral Center for Cardiac Amyloidosis Mondor Amyloidosis Network GRC Amyloid Research Institute and Cardiology Department APHP Henri Mondor Hospital Créteil France; ^4^ Institut Universitaire de Cardiologie et de Pneumologie Université Laval Québec Canada; ^5^ Department of Medical Imaging Henri Mondor Hospital APHP Créteil France; ^6^ Department of Nuclear Medicine Henri Mondor University Hospital APHP Créteil France; ^7^ Université Paris Est Créteil Créteil France; ^8^ Department of Hepatology Henri Mondor University Hospital APHP Créteil France; ^9^ Department of Cardiology Amiens University Hospital Amiens France; ^10^ INSERM Unit U955 Clinical Epidemiology and Ageing (CEpiA) Paris‐Est Créteil University, Val‐de‐Marne Créteil France

**Keywords:** cardiac amyloidosis, carpal tunnel syndrome, aortic stenosis

## Abstract

**Background:**

The frequent association between transthyretin wild‐type (TTRwt) cardiac amyloidosis (CA) and aortic stenosis (AS) suggests a bidirectional relationship: TTRwt‐CA could induce AS and vice versa. Systemic manifestations may highlight this interaction: systemic amyloidogenesis would lead to systemic symptoms, CA, and AS, whereas the myocardial stresses induced by degenerative AS might promote local amyloidogenesis without systemic symptoms. Carpal tunnel syndrome (CTS) is the most frequently reported extracardiac symptom. Through a comparison of TTRwt‐CA patients with and without CTS, we sought to determine whether CTS serves as a reliable indicator of systemic involvement and its impact on cardiac and valvular characteristics.

**Methods and results:**

A total of 411 consecutive patients with TTRwt‐CA were included. CTS, present in 70.3%, was associated with a younger age (80 vs. 84 years, *p* < 0.001), more extracardiac symptoms, and advanced CA, with greater cardiac remodeling and a higher heart‐to‐mediastinum ratio (1.63 vs. 1.54; *p* = 0.012) compared to patients without CTS. AS was present in 21% and 31% of patients with and without CTS, respectively (*p* = 0.024). Except for AS, these associations remained significant after adjusting for confounding factors. In severe AS, patients with CTS exclusively exhibited low‐flow low‐gradient (LFLG) AS and less severe class of aortic valvular calcium score (5.6% vs. 60%; *p* = 0.006) compared to those without CTS.

**Conclusion:**

Our findings suggest that CTS may delineate two phenotypes in TTRwt‐CA: a systemic phenotype associated with advanced CA and poorly calcified LFLG AS, and a cardiac phenotype characterized by less severe CA and a mixed pattern of highly calcified AS, suggesting disparate pathophysiologies.

## Graphical abstract

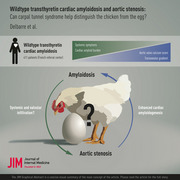

**Abbreviations**
AS: aortic stenosis
ATTRwt: wild‐type transthyretin amyloidosis
AVCS: aortic valvular calcium score
CA: cardiac amyloidosis
CTS: carpal tunnel syndrome
LCS: lumbar canal stenosis surgery
LFLG: low‐flow low‐gradient
TTRwt: wild‐type transthyretin

## Introduction

Wild‐type transthyretin amyloidosis (ATTRwt) is a systemic disorder characterized by the pathological accumulation in various organs of insoluble amyloid fibrils composed of misfolded wild‐type transthyretin (TTRwt) [1]]. However, the clinical relevance of these deposits varies, as evidenced by their presence in the hearts of up to 25% of octogenarians [2], whereas overt cardiac dysfunction, defining cardiac amyloidosis (CA), is much less prevalent, affecting only 2%–3% of individuals over 75 years of age [3].

Beyond age and male gender [4], the factors enhancing amyloidogenesis remain elusive. An intriguing avenue of exploration stems from the observed association between CA and aortic stenosis (AS). Although some instances may be coincidental, given their predominant occurrence in elderly men, the high frequency of CA in AS, rising up to 16% in severe AS undergoing transcatheter aortic valve replacement [5], suggests a potential causal link, which could be bidirectional: Amyloidosis might contribute to AS development and vice versa [6, 7].

However, unraveling this cause‐and‐effect relationship is challenging. A key aspect that could offer insight into this association is the presence of systemic symptoms. Indeed, to develop further the complex interplay between AS and CA, on the one hand, a systemic process of amyloid accumulation is expected to manifest as systemic symptoms of the amyloid spectrum, such as carpal tunnel syndrome (CTS), CA, and concomitant AS. In this scenario, amyloidosis may contribute to the development or progression of AS through inflammation, endothelial dysfunction, and/or direct deposition of amyloid fibrils within the aortic valve [8]. On the other hand, myocardial pressure overload‐induced by “conventional” degenerative AS might trigger molecular pathways within the myocardium that promote amyloid fibril formation and deposition, leading to the development of CA, even in the absence of significant systemic symptoms.

Therefore, we hypothesize that systemic symptoms may impact both cardiac and AS features. Given that not all systemic symptoms are routinely assessed, our focus was on CTS, which is the most commonly reported extracardiac symptom and easily identifiable. Hence, we compare the extracardiac and cardiac phenotypes of patients with and without CTS to ascertain whether CTS could serve as a reliable indicator of systemic involvement in ATTRwt and how the presence of CTS influences both cardiac involvement and the phenotype of AS.

## Methods

### Population and definitions

**Fig. 1:**
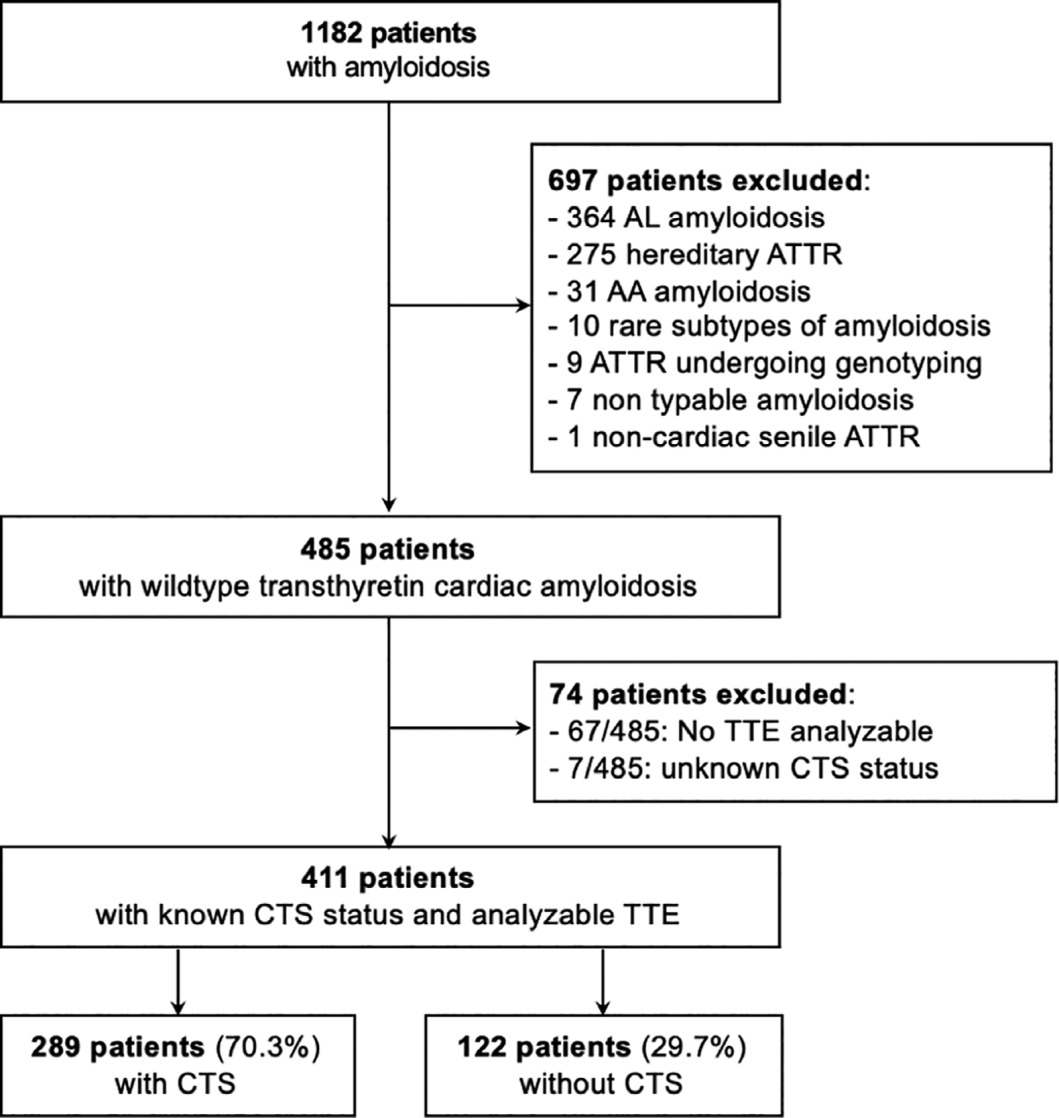
Flowchart of the study. AL, light chain amyloidosis; ATTR, transthyretin amyloidosis; CTS, carpal tunnel syndrome; TTE, transthoracic echocardiography.

We retrospectively analyzed the data of 1182 patients with CA of diverse subtypes recruited prospectively between 2012 and 2021 in the CA French registry of the referral center for CA at Henri Mondor Hospital. The inclusion criteria were a proven TTRwt‐CA. Exclusion criteria were unanalyzable baseline transthoracic echocardiography and unknown CTS status (Fig. 1).

The TTRwt‐CA was defined by strong cardiac retention on bone scintigraphy (with technetium‐labeled radiotracers) in the absence of gammopathy or by TTR amyloid deposits on a biopsy sample without pathogenic TTR mutation [9]. The presence of extracardiac symptoms currently considered relevant for systemic amyloidosis was systematically assessed for each patient during the inclusion visit, using a preestablished data collection grid. Ten extracardiac features were retained in the analysis: history or symptoms of CTS (with or without surgery) [10], history of lumbar canal stenosis surgery (LCS) [11], hearing loss [12], peripheral neuropathy symptoms [13, 14], orthostatic hypotension [15], gastro‐intestinal dysautonomia symptoms [16], purpura, periorbital ecchymosis, macroglossia, and ungual dystrophy [17].

### Reviewing of echocardiography and other imaging

Baseline transthoracic echocardiography for each included patient was reviewed for a second analysis by an experienced cardiologist (MSA), blind to the initial report, in order to homogenize the measures for AS and to assess parameters not performed routinely.

Left ventricle ejection fraction was abnormal if <50%, severe interventricular septal hypertrophy if interventricular septal thickness if ≥16 mm for women and ≥17 mm for men [18], posterior wall hypertrophy if posterior wall thickness ≥13 mm for women and ≥14 mm for men [19], asymmetric hypertrophy if interventricular septal thickness to posterior wall thickness ratio >1.3 [20], and restrictive filling pattern if *E*/*A* transmitral flow velocities >2 [21].

Aortic flow was recorded using continuous‐wave Doppler from several views (apical 5‐chamber, right parasternal, and suprasternal). AS was defined by the presence of *V*_peak_ > 2 m/s and was then graded as mild, moderate, and severe regarding ACC/AHA current guidelines [22]. When aortic valve area was <1 cm^2^ or 0.6 cm^2^/m^2^, AS was classified by flow‐gradient pattern: High‐gradient AS was defined by a mean gradient ≥40 mmHg; low‐flow low‐gradient (LFLG)‐AS by a mean gradient <40 mmHg, an indexed stroke volume <35 mL/m^2^, low‐gradient normal‐flow by a mean gradient <40 mmHg, and an indexed stroke volume ≥35 mL/m^2^ [22, 23]. High‐gradient and LFLG‐AS were considered severe, whereas low‐gradient normal‐flow AS were considered moderate [24, 25].

When available, the chest computed tomographic sequence associated with the ^99m^Tc bone scintigraphy at inclusion was reviewed by an expert radiologist (RN) to determine the aortic valvular calcium score (AVCS) in patients with aortic valve area <1 cm^2^ or <0.6 cm^2^/m^2^. Then, the AVCS was split into severity classes defined as follows [23, 26]: severe AVCS when ≥2000 AU for men and ≥1200 AU for women, moderate AVCS when between 800 and 2000 AU for men and 400 and 1200 AU for women, and mild AVCS when <800 AU for men and <400 AU for women.

### Statistical analysis

Baseline variables of patients with and without CTS were compared. Continuous variables were expressed as median (interquartile range) and were compared using Wilcoxon rank‐sum test. Proportions were expressed as percentages and were compared using *χ* ^2^ test, or Fisher exact test. A Cochran–Armitage trend test was used to examine the relationship between CTS characteristics, treated as an ordinal variable (no CTS > unilateral CTS > bilateral CTS), and binary variables representing systemic manifestations and cardiac features.

To investigate the association of CTS with systemic and cardiac variables while accounting for potential confounding factors, a multivariate logistic regression model was employed. CTS was used as the dependent variable, and the association was tested successively for each variable significantly associated with CTS (*p* < 0.05) in univariate analysis, adjusting for confounding factors as covariates. The following variables were used as possible confounders: age, gender, diabetes mellitus, and body mass index for extracardiac manifestations, and age, gender, diabetes mellitus, body mass index, and arterial hypertension for cardiac features, except for severe interventricular septum hypertrophy and posterior wall hypertrophy, for which gender was excluded from the model as it was already considered in the variable definition. Statistics were performed using STATA software, version 18.0. To study the correlation between cardiac and extracardiac variables, we used the “*corrr*” package in the “R” software. This package allowed us to calculate the correlation coefficients between each pair of variables. To visualize these correlations, we employed “*factoextra*” and “*ggplot2*” packages to perform an unsupervised hierarchical clustering of the correlation coefficients. The results were then modeled into a dendrogram, which graphically represents the clustering of variables based on their correlation. This approach helps to identify groups of variables that are closely related to each other. This investigation was in line with the Declaration of Helsinki.

The study was approved by the local institutional review board (Hôpital Henri‐Mondor, Creteil, France: authorization numbers 1431858 and registered with the French National Data Protection Commission (Commission nationale de l'informatique et des libertés, Paris, France; authorization number 2215384 v 0).

## Results

### Baseline characteristics

Our final cohort consisted of 411 patients (Fig. 1, Table 1), including 289 with CTS (70.3%) and 122 without (29.7%). CTS was bilateral for 216/289 cases (74.7%) and preceded CA diagnosis of 9.2 years [11.7].

**Table 1:** Comparison of the baseline characteristics regarding carpal tunnel syndrome (CTS) status

	Patients with CTS	Patients without CTS	
*N*	(*n* = 289)	(*n* = 122)	*p*‐value
**Demographic features**			
Age at diagnosis (years)	80.0 [9.5]	83.6 [7.3]	**<0.001**
Male gender, *n* (%)	252 (87.2)	103 (84.4)	*0.454*
Height (cm)	170 [10]	170 [14]	*0.384*
Weight (kg)	72 [16]	70 [15]	*0.070*
Body mass index (kg/m^2^)	24.8 [3.9]	24.7 [4.2]	*0.152*
**Cardiovascular risk‐factors**			
Hypertension, *n* (%)	170 (58.8)	75 (61.5)	*0.617*
Diabetes mellitus, *n* (%)	50 (17.3)	20 (16.4)	*0.823*
Obesity, *n* (%)	32 (11.1)	9 (7.4)	*0.253*
Dyslipidaemia, *n* (%)	110 (38.1)	45 (36.9)	*0.822*
Previous or current smokers, *n* (%)	82 (28.4)	32 (26.2)	*0.657*
**Baseline clinical features**			
Cardiac manifestations			
Heart rate (beats/min)	72 [15]	74 [15]	*0.670*
Systolic blood pressure (mmHg)	127 [30]	130 [25]	*0.451*
Diastolic blood pressure (mmHg)	74 [14]	74 [18]	*0.560*
Orthostatic hypotension symptoms, *n* (%)	83 (28.7)	35 (28.7)	*0.995*
NYHA III–IV vs. I–II, *n* (%)	105 (36.3)	48 (39.3)	*0.564*
Prior pacemaker or ICD implantation, *n* (%)	49 (17.0)	22 (18.0)	*0.792*
Prior aortic valve replacement for AS, *n* (%)	10 (3.5)	6 (4.9)	*0.485*
Coronary arteries diseases, *n* (%)	80 (27.7)	31 (25.4)	*0.636*
Extracardiac manifestations, *n* (%)			
History of lumbar canal stenosis surgery, *n* (%)	44 (15.2)	2 (1.6)	**<0.001**
Hearing loss symptoms, *n* (%)	179 (61.9)	52 (42.6)	**<0.001**
Periorbital ecchymosis, *n* (%)	7 (2.4)	6 (4.9)	*0.187*
Macroglossia, *n* (%)	15 (5.2)	9 (7.4)	*0.388*
Purpura, *n* (%)	14 (4.8)	5 (4.1)	*0.914*
Ungual dystrophy	49 (17.0)	14 (11.5)	*0.159*
Peripheral neuropathy symptoms, *n* (%)	118 (40.8)	27 (22.1)	**<0.001**
Gastro‐intestinal dysautonomic symptoms, *n* (%)	75 (26.0)	27 (22.1)	*0.413*
**Pathologic features**			
Amyloid deposits on salivary glands biopsy, *n* (%)	92 (31.8)	27 (22.1)	**0.048**
**Biological features**			
Serum creatinine (µmol/L)	108 [42]	111 [45]	*0.051*
eGFR (mL/min/1.25 m^2^)	63.2 [25.4]	59.8 [23.4]	**0.033**
NT‐proBNP (ng/L)	3070 [3701]	3705 [5568]	**0.015**
HS troponin (ng/L)	64 [42]	70 [66]	**0.028**
Hemoglobin (g/dL)	13.4 [2.2]	13.3 [2.4]	*0.340*
**Electrocardiogram**			
Sinus rhythm, *n* (%)	162 (56.1)	72 (59.0)	*0.588*
Atrial fibrillation, *n* (%)	115 (39.7)	39 (31.2)	*0.134*
Atrioventricular block, *n* (%)	91 (31.5)	32 (26.2)	*0.288*
Stimulation, *n* (%)	18 (6.2)	12 (9.8)	*0.215*
PR interval duration (ms)	200 [61]	200 [60]	*0.670*
QRS interval duration (ms)	120 [48]	110 [54]	*0.789*
Low voltage, *n* (%)	70 (24.2)	24 (19.7)	*0.316*

*Note*: Values are *n* (%) or median [IQR], and proportions were compared with *χ* ^2^ test or Fisher exact test when appropriate.

Abbreviations: AS, aortic stenosis; eGFR, estimated glomerular filtration rate (MDRD equation); HS troponin, high sensitivity cardiac troponin; ICD, implantable cardioverter‐defibrillator.

Bold values significant analyses and italics are non‐significant analyses.

In univariate analysis and compared to patients without CTS, patients with CTS (Table 1) were significantly younger (*p* < 0.001), had a comparable distribution of men, cardiovascular risk factors, and cardiac and electrocardiographic manifestations but had more extracardiac manifestations of the ATTRwt spectrum, including history of LCS (*p* < 0.001), hearing loss (*p* < 0.001), and peripheral neuropathy (*p* < 0.001), and had more frequently amyloid deposits in accessory salivary gland biopsy (*p* = 0.048). The cutaneous manifestations usually found in light‐chain amyloidosis were not associated with CTS. Regarding biologic findings, CTS was associated with a higher estimated glomerular filtration rate (*p* = 0.033), lower NT‐proBNP (*p* = 0.015), and high‐sensitivity troponin (*p* = 0.028). Despite a younger age than patients without CTS and a comparable proportion of cardiovascular risk factors (Table 1), patients with CTS had a significant increase in left ventricle remodeling (Table 2), with more severe interventricular septum and posterior wall hypertrophies (*p* = 0.029 and *p* = 0.018, respectively), and an increase in left ventricle posterior wall thickness (*p* = 0.015). In addition to this increased remodeling on transthoracic echocardiography, patients with CTS had an increase in heart‐to‐mediastinum ratio (*p* = 0.0012) on ^99m^Tc bone scintigraphy, compared to patients without CTS. The remaining left ventricle parameters, including left ventricle ejection fraction, *E*/*A* and *E*/*e*′ ratios, and systolic and diastolic diameters or volumes, were comparable between the two groups. Regarding aortic valve parameters, patients with CTS had significantly less AS than patients without CTS (*p* = 0.024).

**Table 2:** Baseline imagery features regarding carpal tunnel syndrome (CTS) status

	Patients with CTS	Patients without CTS	*p*‐value
	(*n* = 289)	(*n* = 122)	
**TTE LV parameters**			
LVEF (%)	49 [18]	51 [18]	*0.320*
LGS (%)	−10.0 [−4.8]	−10.8 [−5.6]	*0.217*
LV EDD (mm)	44 [8]	44 [11]	*0.752*
LV ESD (mm)	32.0 [10.1]	31.0 [11.5]	*0.270*
LV EDV (mL)	83 [42]	83 [38]	*0.445*
LV ESV (mL)	40 [27]	39 [28]	*0.263*
Remodeling parameters			
End‐diastolic IVS thickness (mm)	18 [4]	17 [5]	*0.063*
Severe IVS hypertrophy, *n* (%)	19 6 (64.8)	69 (56.6)	**0.029**
LV end‐diastolic PWT (mm)	17 [4]	16 [4]	**0.015**
PW hypertrophy, *n* (%)	248 (85.8)	93 (76.2)	**0.018**
RWT (mm)	0.77 [0.31]	0.73 [0.28]	*0.051*
Asymmetric hypertrophy, *n* (%)	28 (9.7)	16 (13.1)	*0.305*
Indexed LV mass (g/m^2^)	181 [62]	167 [74]	*0.103*
MCF (%)	25.6 [15.6]	29.5 [14.2]	*0.060*
LV diastolic parameters			
*E*/*A* ratio	2.0 [1.7]	1.2 [2.2]	*0.867*
*E*/*e*′ ratio	16.5 [8.4]	17.08 [8.4]	*0.241*
Aortic stenosis, *n* (%)	60 (20.76)	38 (31.15)	**0.024**
Severe	19 (6.57)	15 (12.30)	*0.054*
Moderate	23 (7.96)	13 (10.66)	*0.377*
Mild	18 (6.23)	10 (8.20)	*0.469*
**Scintigraphy features**			
Heart to mediastinum ratio	1.63 [0.36]	1.54 [0.36]	**0.0012**

*Note*: Values are *n* (%) or median [IQR], and proportions were compared with *χ* ^2^ test or Fisher exact test when appropriate.

Abbreviations: EDD, end‐diastolic diameter; EDV, end‐diastolic volume; ESD, end‐systolic diameter; ESV, end‐systolic volume; IVS, interventricular septum; LGS, longitudinal global strain; LV, left ventricle; LVEF, left ventricular ejection fraction; MCF, myocardial contraction fraction; PW, posterior wall; PWT, posterior wall thickness; RWT, relative wall thickness; TTE, transthoracic echocardiography.

Bold values significant analyses and italics are non‐significant analyses.

**Fig. 2:**
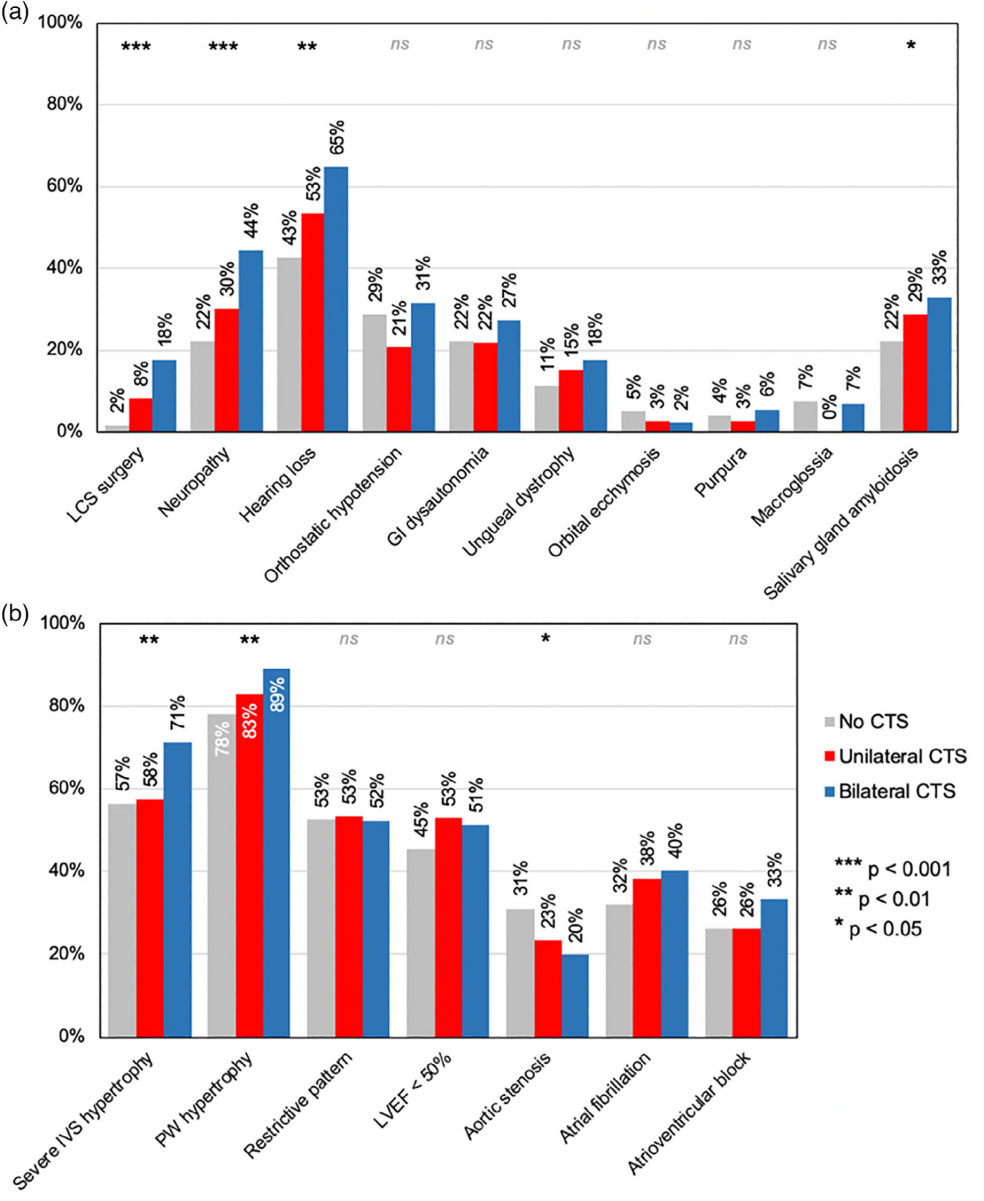
**Prevalence of extracardiac (a) and cardiac features (b) according to the characteristics of the CTS**. Analysis were performed using Cochran–Armitage trend test, with CTS as ordinal variable (no CTS > unilateral CTS > bilateral CTS). AV, atrioventricular; CTS, carpal tunnel syndrome; GI, gastro‐intestinal; IVS, interventricular septum; LCS, lumbar canal stenosis; LVEF, left ventricle ejection fraction; ns, not significant; PW, posterior wall.

A potential “dose effect” on both systemic manifestations and certain cardiac characteristics was observed, contingent upon whether there was no CTS, unilateral CTS, or bilateral CTS: The Cochran–Armitage trend test revealed an upward trend between number of CTS and prevalence of systemic manifestations within the ATTRwt spectrum (Fig. 2a), including LCS (*p* < 0.001), neuropathy (*p* < 0.001), and hearing loss (*p* < 0.01). Conversely, no discernible trend was observed for manifestations typically linked to hereditary ATTR or AL amyloidosis (*p* > 0.05), such as gastro‐intestinal dysautonomia, periorbital ecchymosis, or macroglossia. Concerning cardiac features (Fig. 2b), there was also an upward trend in the proportion of parameters related to left ventricle remodeling, including severe IVS hypertrophy (*p* < 0.01) and posterior left ventricle wall hypertrophy (*p* < 0.01), whereas no trend was evident for diastolic function parameters or electrocardiographic features (*p* > 0.05). Intriguingly, there was a downward trend in the prevalence of AS (*p* < 0.05).

**Fig. 3:**
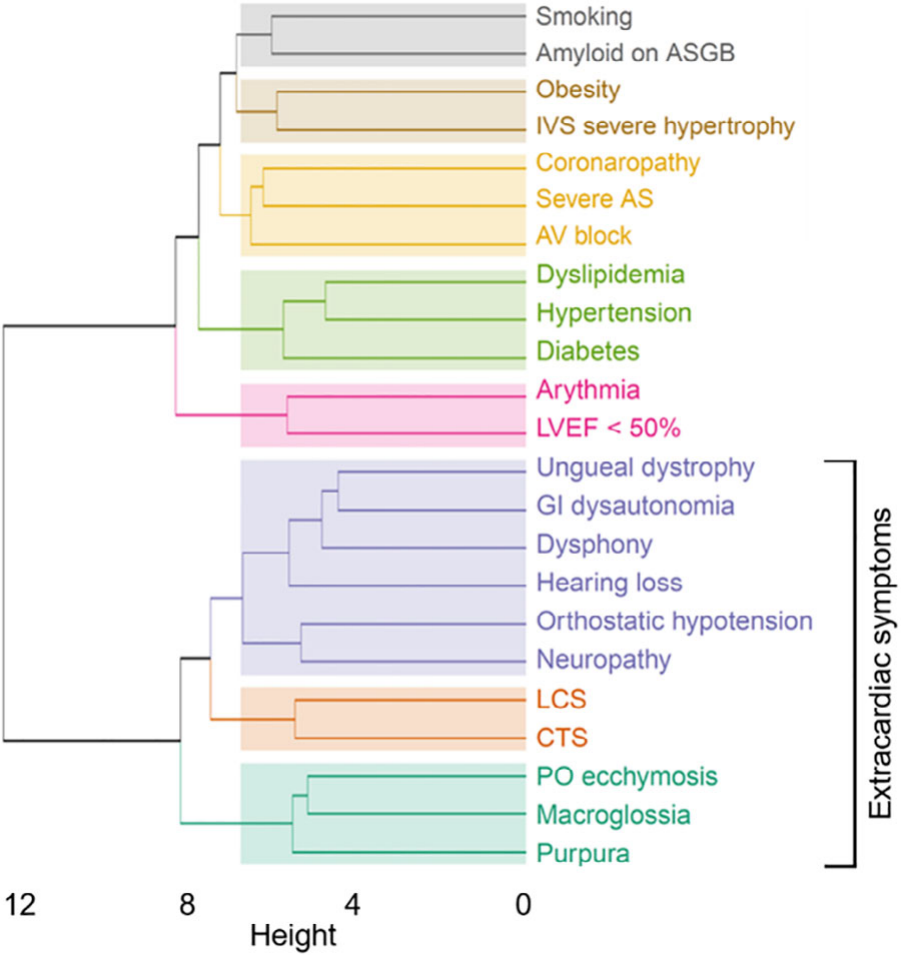
**Hierarchical clustering of extracardiac and cardiac manifestations**. In this dendrogram, based on unsupervised hierarchical clustering of variables correlation, the height at which two branches merge indicates their level of correlation: the lower the heights, the higher the correlation. Closer branches imply stronger relationships between the variables. AS, aortic stenosis; ASGB, accessory salivary gland biopsy; AV, atrioventricular; CTS, carpal tunnel syndrome; GI, gastro‐intestinal; IVS, interventricular septum; LCS, lumbar canal stenosis surgery; LVEF, left ventricle ejection fraction; PO, periorbital.

The various systemic manifestations of the disease correlated with each other, as shown in Fig. 3. Moreover, the correlation between signs was stronger when targeting the same tissue: Tenosynovial manifestations (CTS and LCS) were more correlated with each other, and the same trend was observed for neuropathic (neuropathy and orthostatic hypotension) or cutaneous (purpura, periorbital ecchymosis, macroglossia) manifestations. The dendrogram also showed more intriguing associations, such as smoking history with amyloid deposits in salivary gland biopsy.

In multivariate analysis (Table 3) and after adjustment for significant confounding factors, CTS remained independently associated with LCS (*p* = 0.003), hearing loss (*p *< 0.001), peripheral neuropathy (*p* = 0.004), lower hypersensitive troponin (*p* = 0.022), increased left ventricle posterior wall thickness (*p* = 0.046), posterior wall hypertrophy (*p* = 0.047), and higher heart‐to‐mediastinum ratio (*p* = 0.001). However, there is no longer an association between CTS and AS (*p* = 0.361), NT‐proBNP (*p* = 0.230), severe IVS hypertrophy (*p* = 0.059), or amyloid deposits in salivary gland biopsy (*p* = 0.082), although there is still a trend for the latter two.

**Table 3:** Multivariate analysis to assess the association of carpal tunnel syndrome (CTS) with systemic and cardiac variables

	Adjusted OR [95% CI]^a^	Adjusted *p*‐value^a^
**Extracardiac symptoms**		
History of lumbar canal stenosis surgery	8.71 [2.05–36.96]	**0.003**
Hearing loss symptoms	2.28 [1.45–3.57]	**<0.001**
Peripheral neuropathy symptoms	2.08 [1.26–3.43]	**0.004**
Amyloid deposits on salivary gland biopsy	1.59 [0.95–2.64]	*0.075*
**Biologic features**		
Serum creatinine (µmol/L)	0.99 [0.98–0.99]	**0.032**
NT‐proBNP (ng/L)	1.04 [0.99–1.00]	*0.230*
HS Troponin (ng/L)	0.99 [0.98–0.99]	**0.022**
**Echocardiographic features**		
Severe IVS hypertrophy^b^	1.55 [0.98–2.43]	*0.059*
LV end‐diastolic PWT (mm)	1.07 [1.01–1.15]	**0.046**
Posterior wall hypertrophy^b^	1.77 [1.01–3.09]	**0.047**
Aortic stenosis	0.80 [0.48–1.32]	*0.361*
**Scintigraphic features**		
Heart to mediastinum ratio	8.8 [2.1–36.3]	**0.001**

*Note*: CTS was used as the dependent variable, and the association was tested sequentially for each variable significantly associated with CTS (*p *< 0.05) in univariate analysis, adjusting for confounders as covariates.

Abbreviations: HS troponin, high‐sensitivity troponin; IVST, interventricular septum thickness; LV, left ventricle; PWT, posterior wall thickness.

^a^ Associations with extracardiac manifestations were adjusted on age, gender, diabetes mellitus and body mass index. Associations with cardiac parameters were adjusted on and age, gender, diabetes mellitus, body mass index, and arterial hypertension.

^b^ Severe IVS hypertrophy and PW hypertrophy were not adjusted on sex as covariate, since it was already considered in the variable definition (see “Method” section).

Bold values significant analyses and italics are non‐significant analyses.

### Aortic stenosis parameters regarding CTS

Although patients with and without CTS had a similar proportion of AS after adjustment for confounding factors (Table 3), some significant differences were observed in stenosis parameters in the case of severe AS (Table 4). First of all, patients with CTS only had low‐gradient stenosis and no high‐gradient pattern, whereas the latter concerns 33.3% of patients without CTS. In spite of a higher proportion of low‐gradient stenosis in patients with CTS (*p* = 0.024), severity parameters of AS were comparable in both groups, such as indexed aortic surface (*p* = 0.507) or indexed stroke volume (*p* = 0.290). Then, regarding aortic valve parameters on computed tomography, the median AVCS of patients with and without CTS was not significantly different (*p* = 0.114). However, when AVCS was classified according to severity, which depends on gender (see “Method” section), patients with CTS had less severe class of AVCS than those without CTS (*p* = 0.006). Moreover, when patients with high‐gradient AS were removed from the analysis, the proportion of severe AVCS remained lower in patients with CTS compared to patients without CTS (5.6% vs. 42.9%, *p* = 0.022). Finally, as in patients without AS, the heart/mediastinum ratio is higher in patients with CTS compared to patients without CTS (*p* = 0.03).

**Table 4:** Aortic stenosis (AS) parameters of patients with severe AS^a^ regarding presence of carpal tunnel syndrome (CTS)

	Patients with CTS (*n* = 19)	Patients without CTS (*n* = 15)	*p*‐value
**Demographic parameters**			
Age at diagnosis (years)	83.8 [7.6]	84.9 [7.0]	*0.298*
Male gender, *n* (%)	17 (89.5)	12 (80)	*0.600*
Body mass index (kg/m^2^)	25.9 [5.1]	25.1 [4.0]	*0.205*
**TTE AS parameters**			
Left ventricle ejection fraction (%)	50 [17]	51 [19]	*0.886*
Aortic valve area (cm^2^)	0.87 [0.22]	0.80 [0.31]	*0.160*
Indexed aortic valve area (cm^2^/m^2^)	0.48 [0.13]	0.43 [0.22]	*0.507*
Transaortic mean pressure gradient (mmHg)	18 [9]	22 [33]	*0.206*
Peak aortic jet velocity (m/s)	2.8 [0.7]	3.0 [1.9]	*0.199*
Cardiac index (L/min/m^2^)	1.82 [0.60]	2.12 [1.22]	*0.425*
Indexed stroke volume (mL/m^2^)^b^	23.3 [8.3]	27.3 [10.6]	*0.290*
AS patterns			
HG, *n* (%)	0 (0.0)	5 (33.3)	**0.024**
LFLG, *n* (%)	19 (100.0)	10 (66.7)	
Opening limitation of aortic leaflets			
Moderate, *n* (%)	0 (0)	3 (20)	**0.041**
Severe, *n* (%)	19 (100)	12 (80)	
**CT aortic features**			
Available AVCS on CT, *n* (%)	18 (94.7)	10 (66.7)	*0.800*
AVCS (Agatston units)	1201 [956]	2895 [3067]	*0.114*
AVCS severity			
Mild AVCS, *n* (%)	4 (22.2)	1 (10)	**0.006**
Moderate AVCS, *n* (%)	13 (72.2)	3 (30)	
Severe AVCS, *n* (%)	1 (5.6)	6 (60)	
**Scintigraphic features**			
Heart/mediastinum ratio	1.53 (0.21)	1.31 (0.24)	**0.030**

*Note*: Values are *n* (%) or median [IQR]. Proportions were compared with *χ* ^2^ test or Fisher exact test when appropriate.

Abbreviations: AVCS, aortic valvular calcium score; CT, computed tomography; HG, high‐gradient; LFLG, low‐flow low‐gradient; TTE, transthoracic echocardiography.

^a^ Severe AS were defined as LFLG of HG AS.

^b^ Doppler‐derived indexed stroke volume.

Bold values significant analyses and italics are non‐significant analyses.

**Fig. 4:**
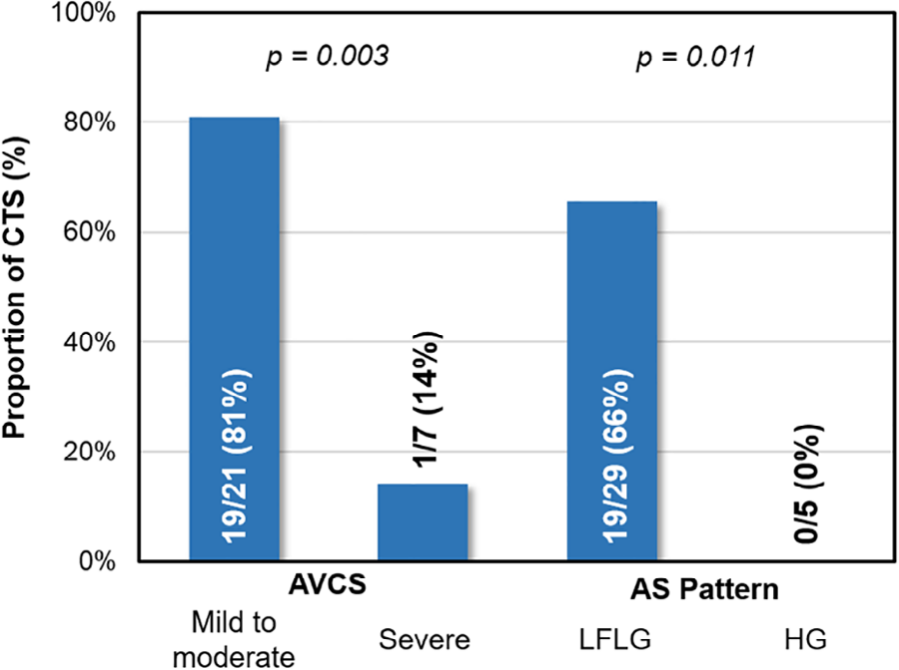
**Proportion of carpal tunnel syndrome (CTS) regarding severity of AVCS (left) and flow‐gradient aortic stenosis (AS)‐pattern (right) in patients with severe AS**. Analysis were performed using Fisher exact test. AVCS, aortic valvular calcium score; HG, high‐gradient; LFLG, low‐flow low‐gradient.

Then, to further explore the possible relationship between severe AS and systemic phenotype, we assessed the proportion of CTS according to the severity of AVCS and flow‐gradient pattern of AS (Fig. 4). This figure suggests that the more calcified the AS or the higher the gradient, the lower the proportion of CTS.

## Discussion

In this study involving 411 patients with TTRwt‐CA, 70.3% had CTS. Compared to those without CTS, patients with CTS were notably younger, presented with more extracardiac manifestations of the ATTRwt spectrum (Table 1), demonstrated elevated left ventricle remodeling parameters alongside a higher heart‐to‐mediastinum ratio, and displayed a lower prevalence of AS (Table 2). In multivariate analysis, after adjusting for age and other relevant confounders (see “Statistical analysis” section), CTS remains associated with extracardiac manifestations, posterior wall hypertrophy, and a heightened heart‐to‐mediastinum ratio, albeit no longer with AS (Table 3). Regarding severe AS characteristics, patients with CTS exclusively exhibited LFLG‐AS, with a significantly lower proportion of severe AVCS compared to patients without CTS (Table 4). Furthermore, patients with severe AVCS had significantly less CTS than those with low to moderate AVCS (Fig. 4).

### Carpal tunnel syndrome: a hallmark for a systemic phenotype of ATTRwt

The strong and independent association of CTS with extracardiac symptoms of the ATTRwt spectrum [12, 13, 16] suggests a systemic phenotype of CA on the one hand and a more cardiac localized on the other one. While confirming the amyloid process behind these symptoms without histology is not feasible, the significance of their association following adjustments for confounding factors (Table 3) and their susceptibility to amyloid burden, reflected by the bilateral occurrence of CTS (Fig. 2), implies a connection to amyloidosis. This systemic phenotype also appears to be associated with more advanced CA as patients with CTS showed more severe left ventricle remodeling, independently of cardiovascular risk factors, and a greater heart/mediastinum ratio [27] on bone scintigraphy compared to those without CTS (Tables 2 and 3), and may explain their worse prognosis [28]. This increase in remodeling, as noted by Milandri et al. [10], could be attributed to the prolonged duration of systemic amyloid deposition, starting from the onset of CTS, a hallmark of diffuse deposition, until the diagnosis of CA, which occurred 9.2 years [11.7] later.

Whether the clinical expression is systemic or more localized to the heart, the comparable proportion of patients with amyloidosis deposits in the salivary gland in both groups in multivariate analysis is intriguing. It probably reflects a similar potential for systemic dissemination of amyloid fibrils, the impact of which on the organ depends on the increase in local amyloidogenesis, under the influence of pro‐aggregating factors. In joint disorders, for example, amyloidogenesis is probably enhanced by mechanical stresses, as suggested by the very high prevalence of amyloid deposits that can reach up to 93% in joints where major mechanical stresses are exerted: knees [29], lumbar vertebrae [11]. In addition, it seems that long‐term mechanical stress could play a role in the development of ATTRwt [30]. In our study, it is therefore interesting to note that the most correlated factor with amyloid deposits in salivary gland was the smoking history (Fig. 3). In this context, the analysis of patients with severe AS is particularly interesting, as it leads to localized stresses on the myocardium, unlike arterial hypertension, for example, whose effects are much more systemic.

### Aortic stenosis and cardiac amyloidosis: carpal tunnel syndrome to distinguish the chicken from the egg?

Regarding the gradient‐flow pattern of AS, none of the patients with CTS had a high‐gradient stenosis, whereas this type of stenosis affected 33% of patients without CTS (*p* = 0.024). The higher cardiac amyloid burden observed in patients with CTS (*p* = 0.03), suggested by the increase in H/M ratio, is possibly responsible for the exclusive presence of the LFLG pattern (Table 4). Concerning AVCS, 17 out of 18 patients with CTS and severe AS had mild to moderate AVCS (<2000 AU for men and <1200 AU for women).

Interpreting the less severe AVCS in these patients is more challenging. The AVCS is currently used to grade the severity of LFLG‐AS [23], which is often overestimated on echocardiography due to incomplete valve opening caused by reduced opening forces. However, the severity thresholds for AVCS have primarily been established to differentiate the severity of high‐gradient AS [26, 31], whose pathophysiology appears closely linked to valvular calcifications [32]. For this type of AS, the diagnostic performance of AVCS, validated in large international cohorts, is excellent [31]. By extension, these severity thresholds have been applied to LFLG, whose pathophysiology is likely different. Although they correctly predict all‐cause mortality in patients with LFLG‐AS [31], their accuracy in determining the hemodynamic severity of the AS seems less robust: A recent multicenter study in 214 patients with LFLG‐AS, evaluated by both dobutamine stress echocardiography and valvular computed tomography, indicated that the severity threshold of the AVCS is neither sensitive nor specific for predicting LFLG‐AS severity [33]. Specifically, 44.3% of patients with severe LFLG‐AS and 43.5% of patients with moderate AS had a high AVCS. In this context, a low AVCS might not necessarily indicate a lower severity of LFLG‐AS but might reflect a different pathophysiology of AS, unrelated to valvular calcification.

Given the high prevalence of ATTRwt in this category of AS, which can reach up to 29% [5], and a growing amount of evidence suggesting that concomitant AS and CA may have a lower AVCS than AS without CA [34, 35, 36, 37], it is reasonable to consider the underlying role of amyloidosis in the low AVCS of patients with CTS. Regarding the common molecular structures between heart valves and musculoskeletal tendons, a well‐known target of amyloid deposition in systemic ATTRwt [38], the frequency of ATTRwt deposits in explanted aortic valves of patients with AS [8], and the significant increase in non‐calcified valvular tissue in patients with LFLG‐AS compared to high gradient‐AS [39], we could speculate that “low calcified” AS in patients with CTS is the result of the valvular infiltration by amyloid deposits.

In contrast to the profile of patients with CTS, who exhibit a high cardiac and systemic amyloid burden and low‐calcified LFLG‐AS, indicative of amyloid‐related and/or pseudo‐severe AS, patients without CTS presented a different profile. They had fewer systemic signs, a lower cardiac amyloid burden, and more diverse stenosis patterns, often characterized by heavy calcification. Although it might be argued that “conventional” calcified AS could have prompted the earlier detection of CA, most studies indicate that the extracardiac symptoms precede cardiac involvement [40, 41]. The low proportion of systemic manifestations of the amyloid spectrum in these patients with highly calcified AS (Fig. 4) therefore raises the question of the causal role of calcified AS in this preferentially cardiac amyloidogenesis, although the literature is very limited on this subject. We could therefore speculate about a latent diffuse amyloidogenesis in elderly [2], enhanced specifically in myocardium by AS‐induced pressure overload and its subsequent molecular dysregulations, such as plasminogen pathway [42].

It may seem paradoxical that patients with the highest AVCS have the lowest cardiac uptake on scintigraphy. However, unlike the AVCS, which reflects the amount of valvular macrocalcifications, cardiac uptake is likely more related to amyloid deposits than to myocardial microcalcifications. Although the question is not settled [43], a recent report supports this point: Three patients spontaneously recovered from their ATTRwt by developing polyclonal anti‐transthyretin antibodies [44]. The simplest and most logical explanation for the multimodal improvement of cardiac parameters, including cardiac uptake on bone scintigraphy, is the reduction of amyloid burden mediated by anti‐ATTR antibodies rather than the reduction of calcium deposits, which the antibodies likely do not affect.

If the hypotheses we formulated were confirmed by further research, it could have significant clinical and therapeutic implications. From a diagnostic perspective, the identification of a non‐severe AVCS in the context of LFLG‐AS could serve as a red flag, prompting the investigation for ATTRwt, especially when systemic signs are present. As approximately 50% of low‐gradient AS cases have a non‐severe AVCS [31], this approach might detect a significant number of patients with ATTRwt, leading to earlier diagnosis. From a therapeutic perspective, this first raises the question of the effectiveness of specific treatments for ATTRwt, such as stabilizers, in slowing the progression of amyloid AS. Conversely, it also questions the potential benefit of early aortic valve replacement in patients with conventional stenosis to prevent the development of TTRwt‐CA.

### Limitations

Our study has several biases, primarily related to patient selection. We excluded 75 out of 485 patients: 67 (13.8%) due to unanalyzable echocardiography loops and 7 (1.4%) due to unknown CTS status. Although this number of exclusions is not unusual for a retrospective study, it raises concerns about the representativeness of the analyzed population. However, the absence of an echocardiography loop leading to exclusion was likely due to storage issues of the loop rather than patient characteristics. Therefore, its impact on the analysis is probably limited. To support this point, we compared the baseline characteristics (demographic, cardiovascular risk factors, cardiac biomarkers) of included and excluded patients and found no significant differences.

The second and major issue of this study is the small sample size in the subgroup of patients with severe AS. This reduction in sample size has significant implications for our findings. First, it inherently reduces the statistical power of the analysis. Although reduced power increases the risk of Type II errors (failing to detect a difference when one truly exists), it does not invalidate the significant differences that are observed. Although the observed significant differences indicate a sufficiently strong effect to be detected despite the limited power, the main concern is the inability to adjust for potential confounding factors that can influence AS parameters, such as age and sex for AVCS.

Finally, although the retrospective design allows for the identification of significant associations that support our initial hypotheses, it does not establish hypothetical causal links.

These limitations highlight the need for a cautious interpretation of our results and emphasize the importance of further studies in patients with AS and TTRwt‐CA. Larger multicenter studies could be conducted to confirm our findings, allowing for adjustments for confounding factors and providing more generalizable data from diverse patient populations. Then, to support the hypothesis that a low AVCS does not exclude severe AS in the setting of systemic ATTR, it is crucial to rule out pseudo‐severe AS using dobutamine stress echocardiography in patients with low AVCS. Third, establishing a causal link between TTRwt‐CA and hypothetical amyloid AS could be achieved by examining explanted valves from patients with systemic ATTR. However, as the advent of transcatheter aortic valve replacement, mechanical valve replacements have become rare in elderly patients, limiting the availability of explanted aortic valves. In this context, autopsy studies may provide valuable data. Finally, to explore the causal relationship between conventional calcified stenosis and localized CA, murine models of ATTR could be useful to study the impact of experimentally induced stenosis on cardiac amyloidogenesis. These research avenues could provide a comprehensive understanding of the interplay between AS and ATTR.

## Conclusion

Our study sheds light on the intricate connection among CTS, CA, and AS. CTS seems a reliable marker for systemic phenotype of ATTRwt, which was associated with more advanced CA, LFLG‐AS, and a less severe AVCS, possibly reflecting amyloid infiltration of the aortic valve. In contrast, patients without CTS had few systemic symptoms, with less severe CA, and mixed phenotype of highly calcified AS, suggesting a preferential cardiac amyloidogenesis, possibly enhanced by a “conventional” AS. Although the results should be interpreted with caution due to the lack of adjustment for potential confounding factors related to the small number of patients with severe AS, these findings prompt important questions about the causal link between AS and ATTRwt, with potential implications for clinical practice and treatment strategies. Further research is needed to validate these findings and understand the underlying mechanisms that could lead to better diagnostic and management approaches for patients with these overlapping conditions.

## Author contributions

**Marc‐Antoine Delbarre**: Conceptualization; methodology; investigation; formal analysis; writing—original draft; writing—review and editing; visualization; software. **Gagan Deep Chadha**: Methodology; writing—review and editing; supervision; validation; investigation. **Mohamed‐Salah Annabi**: Formal analysis; investigation. **Refaat Nouri**: Formal analysis; software. **Amira Zaroui**: Methodology; software; formal analysis. **Paul Blanc‐Durand**: Software; formal analysis. **Diana Rasolonirina**: Software; formal analysis. **Mounira Kharoubi**: Resources; project administration; visualization; data curation. **Ancuta Bejan**: Methodology; formal analysis. **Arnaut Galat**: Methodology; conceptualization. **Silvia Oghina**: Methodology; writing—review and editing; investigation. **Philippe Pibarot**: Methodology; validation. **Christophe Tribouilloy**: Methodology; validation; writing—review and editing; supervision. **Thibaud Damy**: Methodology; validation; investigation; supervision; visualization; writing—review and editing; resources; conceptualization.

## Conflict of interest statement

The author declares no conflicts of interest.

## Funding information

None of the authors received financial support for the research, authorship, and publication of this article.

## Data Availability

Research data are not shared.
